# Discrimination of *Dendrobium* *officinale* and Its Common Adulterants by Combination of Normal Light and Fluorescence Microscopy

**DOI:** 10.3390/molecules19033718

**Published:** 2014-03-24

**Authors:** Chu Chu, Huimin Yin, Li Xia, Dongping Cheng, Jizhong Yan, Lin Zhu

**Affiliations:** 1 College of Pharmaceutical Science, Zhejiang University of Technology, Hangzhou 310014, China; E-Mails: chuchu@zjut.edu.cn (C.C.); 552444577@qq.com (H.Y.); chengdp@zjut.edu.cn (D.C.); 2 Guangdong Food and Drug Vocational College, Guangzhou 510520, China; E-Mail: xiali516@126.com; 3 School of Chinese Medicine, Hong Kong Baptist University, Hong Kong Special Administrative Region, Hong Kong China; E-Mail: linzhu912@gmail.com

**Keywords:** discrimination, *Dendrobium officinale*, adulterants, normal light microscopy, fluorescence microscopy

## Abstract

The stems of *Dendrobium officinale* Kimura et Migo, named *Tie-pi-shi-hu*, is one of the most endangered and precious species in China. Because of its various pharmacodynamic effects, *D. officinale* is widely recognized as a high-quality health food in China and other countries in south and south-east Asia. With the rising interest of *D. officinale*, its products have a high price due to a limited supply. This high price has led to the proliferation of adulterants in the market. To ensure the safe use of *D. officinale*, a fast and convenient method combining normal and fluorescence microscopy was applied in the present study to distinguish *D. officinale* from three commonly used adulterants including *Zi-pi-shi-hu* (*D. devonianum*), *Shui-cao-shi-hu* (*D. aphyllum*), *Guang-jie-shi-hu* (*D. gratiosissimum*). The result demonstrated that *D. officinale* could be identified by the characteristic “two hat-shaped” vascular bundle sheath observed under the fluorescence microscopy and the distribution of raphides under normal light microscopy. The other three adulterants could be discriminated by the vascular bundle differences and the distribution of raphides under normal light microscopy. This work indicated that combination of normal light and fluorescence microscopy is a fast and efficient technique to scientifically distinguish *D. officinale* from the commonly confused species.

## 1. Introduction

The genus *Dendrobium*, containing of 1,100 species or more all over the world, is one of the largest groups of the family Orchidaceae. There are 76 species of *Dendrobium* in China, including 74 species and two varieties [1], of which the dried stem of *D. officinale* is listed in the Chinese pharmacopoeia under the name of *Dendrobii Officinalis Caulis* (*Tie-pi-shi-hu*) as an individual entry [2]. According to Traditional Chinese Medicine theory, the main function of *D. officinale* is to nourish yin and clear away heat-evil, tonifying the stomach and promoting fluid [2]. It was used for maintaining stomach tonicity, promoting body fluid production, and relieving symptoms such as throat dryness and dry eyes with blurred vision in the clinic, and was found to possess immunostimulating [3,4,5] and antitumor effects [6].

Because of its various pharmacodynamic effects, *D.* *officinale* is widely recognized as a high-quality health food in China and other countries in south and south-east Asia. It was reported that in Zhejiang Province the industrial output value of *D. officinale* was up to 2 billion yuan in 2011, and expected to increase to 4 billion yuan in 2015. However, as one of the most endangered and precious Traditional Chinese Medicines, the limited supply and the huge demand for *D. officinale* has triggered a dramatic rise in price. In 2013, the price of dried stems of *D. officinale* was around ¥ 1000–80,000/kg, while other *Dendrobium* species which possess similar morphological and anatomical characteristics to *D.* *officinale*, such as *D.* *devonianum*, *D.* *aphyllum* and *D.* *gratiosissimum*, have much lower prices (less than one-fourth of the price of *D. officinale*). Consequently, adulterants, confused species, and counterfeits have proliferated in the market due to the great disparity in price between them [7,8], making identification of authenticity important and essential to the quality control of *D.* *officinale* and its related products.

Up to now, the authentication and discrimination of *D. officinale* have already been carried out by determination of chemical data [9,10], infrared spectrometry analysis [11], or using molecular biology techniques, such as DNA sequencing [12,13]. However, the previous methods are inadequate because they are either lack a well-accepted marker compound or are complicated to perform, costly, unstable, and time-consuming.

In contrast, as a facile and inexpensive technique, microscopy has been proposed to authenticate *D. officinale* [14,15,16], but the conventional microscopic authentication only provided simple descriptions, and the hand-drawn pictures of some microscopic features could not effectively differentiate the plant of *Dendrobium* species. Nowadays, fluorescence microscopy has earned a special place in the area of scientific research. Fluorescence microscopy is a fast and simply method, which allows less-experienced personnel to identify botanical raw materials. The combination of normal light and fluorescence microscopic technique, which enhanced the accuracy and convenience of identification, has been successfully applied in authentication and quality evaluation of Chinese herbal medicines [17,18,19,20]. The present study focuses on the microscopy technique, combining normal light and fluorescence microscopy, for discrimination of *D.* *officinale* and its commonly adulterants, including *D. devonianum*, *D.* *aphyllum* and *D.* *gratiosissimum*. The color figures of their microscopic characters and related descriptions are presented in detail and compared to distinguish them from each other.

## 2. Results and Discussion

### 2.1. Macroscopic Characters

#### 2.1.1. Stems of *D. officinale*

**Figure 1:**
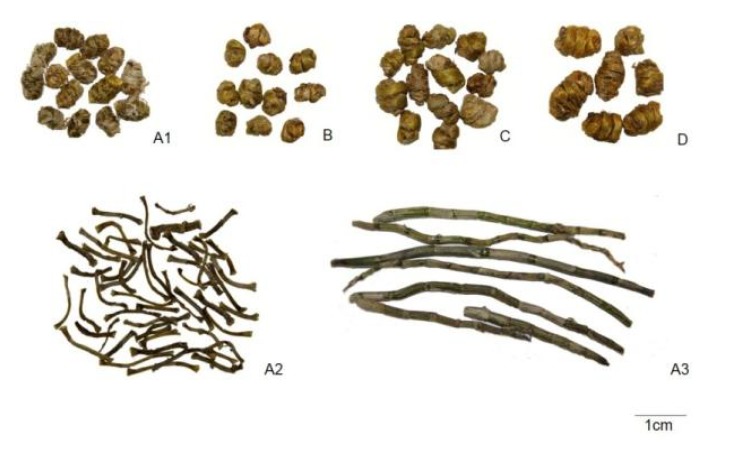
Photographs of four species of *Dendrobium* stems and forms. (**A**) *D. officinale* (**B**) *D. devonianum* (**C**) *D.* *aphyllum* (**D**) *D. gratiosissimum.* 1 *Feng-dou* (dried), 2 Dried samples, 3 Fresh samples.

*Tie-pi-feng-dou*: A commonly trade form of dried stem of *D.* *officinale*. Twist or spring-like, usually 2–6 spiral striation. When stretched, stem of 3.5–8 cm in length, 0.2–0.4 cm in diameter. Outer surface yellowish-green or golden-yellow, marked with fine longitudinal grooves. Node obvious, rudimental grayish-white leaf sheath sometimes can be found in node. Solid, breaks easily; broken surface is flat, grayish-white to grayish green, and slightly horny. Odour: faint; taste: mild; sticky when chewed (Figure 1A1).

*Tie-pi-shi-hu*: dried stem of *D. officinale*, slender and cylindrical, various in length (Figure 1A2).

Fresh *Tie-pi-shi-hu*: cylindrical or flat cylindrical, about 15 cm in length, 0.3–0.8 cm in diameter. Outer surface grayish-green, marked with longitudinal grooves. Texture soft and flexible, broken surface slightly fibrous. Odour: faint; taste: succulent; sticky when chewed (Figure 1A3).

#### 2.1.2. Stems of *D. devonianum*

*Zi-pi-shi-hu* (or *Zi-pi-feng-dou*): Trade form of dried stem of *D. devonianum*. Macroscopic characters are very similar to *Tie-pi-feng-dou* (Figure 1B). Twist or spring-like, usually 2–7 spiral striation. Stem 0.2–0.7 cm in diameter. Outer surface dark greenish-yellow, sometimes with purple spots, marked with fine longitudinal grooves. Node obvious, rudimental grayish-white leaf sheath sometimes can be found in node. Fibrous, difficult to break, broken surface uneven. Odour: faint; taste: mild; sticky when chewed.

#### 2.1.3. Stems of *D. aphyllum*

*Shui-cao-shi-hu* (or *Shui-cao-feng-dou*): Trade form of dried stem of *D. aphyllum*. Appearance is very similar to *Tie-pi-feng-dou* (Figure 1C). Twist or spring-like, usually 2–5 spiral striations. Stems 0.2–1.1 cm in diameter. Outer surface dark yellowish-green, marked with fine longitudinal grooves. Node obvious, rudimental grayish-white leaf sheath sometimes can be found in node. Fibrous, difficult to break, broken surface uneven. Odour: faint; taste: slightly bitter, and not sticky when chewed.

#### 2.1.4. Stems of *D. gratiosissimum*

*Guang-jie-shi-hu* (or *Guang-jie-feng-dou*): Trade form of dried stem of *D.* *gratiosissimum*. Appearance is very similar to *Tie-pi-feng-dou* (Figure 1D). Twist or spring-like, usually with 2–5 spiral striations. Stem 0.2–0.8 cm in diameter. Outer surface dark greenish-yellow, marked with fine longitudinal grooves. Nodes obvious. Fibrous, difficult to break, broken surface uneven. Odour: faint; taste: slightly bitter, and not sticky when chewed.

### 2.2. Microscopic Characters

Characteristic microscopic differences of the four *Dendrobium* species in transverse section of stems are summarized in Table 1, Table 2, Table 3, Table 4, Table 5 and Table 6.

#### 2.2.1. Transverse Section of Stems (Observed Under Normal Light Microscope)

##### 2.2.1.1. Stems of *D. officinale*–Outline is nearly circular (Table 1 and Table 2)

(1). Epidermis: a row of cells, thin and flat, 17–45 μm in diameter, lateral walls were slightly lignified, covered with yellow to orange cuticles. A layer of pericladium consisting of parenchymatous cells and vascular bundles can be observed outside the epidermis sometimes.
(2). Parenchyma: Parenchymatous cells similar in size, usually smaller near the vascular bundles. Parenchymatous cells containing raphides, starch granules or silica masses can be observed.
(3). Vascular bundles: Closed collateral vascular bundles, 78 (52)–134, arranged in 4–5 whorls in parenchyma, with similar size.
(4). Fiber groups: outside vascular bundles, two hat-shaped, consisting of 9–58 fiber cells, 5–37 μm in diameter. Occasionally, a hat-shaped or a ring-shaped fiber group can be observed.
(5). Raphides: occurring in parenchymatous cells near the epidermis, (10) 52–194 μm in length.
(6). Silica masses: occurring in parenchymatous cells outside the vascular bundles.

##### 2.2.1.2. Stems of *D. devonianum*—Outline is nearly circular (Table 1 and Table 3)

(1). Epidermis: a row of cells, thin and flat, 17–85 μm in diameter, lateral walls were slightly lignified, covered with yellow to orange cuticles. A layer of pericladium consisting of parenchymatous cells and vascular bundles can sometimes be observed outside the epidermis.
(2). Parenchyma: Parenchymatous cells various in size, usually small near the vascular bundles. Parenchymatous cells containing raphides, starch granules or silica masses can be observed.
(3). Vascular bundles: closed collateral vascular bundles, 57–91, various in size.
(4). Fiber groups: outside vascular bundles, hat-shaped, consisting of 7–43 of fiber cells, 7–45 μm in diameter.
(5). Raphides: occurring in parenchymatous cells near the epidermis and vascular bundles, 51–132 μm in length.
(6). Silica masses: occurring in parenchymatous cells outside the vascular bundles.

##### 2.2.1.3. Stems of *D. aphyllum*—Outline is nearly circular (Table 1 and Table 4)

(1). Epidermis: a row of cells, thin and flat, 13–38 μm in diameter, lateral walls were slightly lignified, covered with yellow to orange cuticles. A layer of pericladium consisting of parenchymatous cells and vascular bundles can be observed outside the epidermis sometimes.
(2). Parenchyma: Parenchymatous cells similar in size, usually smaller near the vascular bundles. Parenchymatous cells containing raphides, starch granules or silica masses can be observed.
(3). Vascular bundles: closed collateral vascular bundles, 53–85, with similar size.
(4). Fiber groups: outside vascular bundles, hat-shaped, consisting of 8–47 fiber cells, 5–33 μm in diameter.
(5). Raphides: scattered or in bundles, non-specific raphide distribution, 65–202 μm in length.
(6). Silica masses: occurring in parenchymatous cells outside the vascular bundles.

##### 2.2.1.4. Stems of *D. gratiosissimum*—Outline is nearly circular (Table 1 and Table 5)

(1). Epidermis: a row of cells, thin and flat, 11–35 μm in diameter, lateral walls were slightly lignified, covered with yellow to orange cuticles.
(2). Parenchyma: Parenchymatous cells various in size, usually small near the vascular bundles. Parenchymatous cells containing raphides, starch granules or silica masses can be observed.
(3). Vascular bundles: closed collateral vascular bundles, 57–84 (124), various in size.
(4). Fiber groups: outside vascular bundles, hat-shaped, consisting of 19–66 fiber cells, 6–31 μm in diameter.
(5). Raphides: occurring in parenchymatous cells near vascular bundles, 39–124 μm in length.
(6). Silica masses: occurring in parenchymatous cells outside the vascular bundles.

#### 2.2.2. Transverse Section of Stems (Observed Under Fluorescence Microscope)

*Stems of* *D. officinale*—*Light intensity of dried samples is more strongly than fresh ones*. Observed with excitation filter ex 450–500 nm and DM 505 nm emission filters, the cuticle, wall of epidermal cells and vascular bundles emitted green fluorescence. In vascular bundles, xylem vessels and fiber groups outside emitted green fluorescence. Especially, the “two hat-shaped” fiber groups can be easily observed. Some parenchymatous cells emitted green fluorescence (Table 6). The light intensity is various in different samples. Observed with excitation filter ex 530–590 nm and DM 595 nm emission filters, the cuticle and wall of epidermal cells emitted red fluorescence. The fluorescence of vascular bundles is characteristic, sometimes xylem vessels and fiber groups outside emitted strong red fluorescence, sometimes the content of parenchymatous cell around vascular bundles emit red fluorescence, while xylem vessels and fiber groups outside emit no fluorescence. In addition, some parenchymatous cells emitted red fluorescence (Table 6).

*Stems of* *D. devonianum*-*Light intensity of dried samples is more strong than for fresh ones*. Observed with excitation filter ex 450–500 nm and DM 505 nm emission filters, only “hat-shaped” fiber groups can be easily observed. The other fluorescence characteristics of *D. devonianum* are similar to those of *D. officinale* (Table 6). Observed with excitation filter ex 530–590 nm and DM 595 nm emission filters, the fluorescence characteristics of *D. devonianum* are similar to those of *D. officinale* (Table 6).

*Stems* *of* *D. aphyllum*-*Light intensity of dried samples is more strongly than fresh ones.* Observed with excitation filter ex 450–500 nm and DM 505 nm emission filters, the fluorescence characteristics of *D. aphyllum* are nearly the same as those of *D. devonianum* (Table 6). Observed with excitation filter ex 530–590 nm and DM 595 nm emission filters, the fluorescence characteristics of *D. aphyllum* are similar to those of *D. officinale* (Table 6).

*Stems* *of* *D. gratiosissimum*-*Light intensity of dried samples is more strongly than fresh ones*. Observed with excitation filter ex 450–500 nm and DM 505 nm emission filters, the fluorescence characteristics of *D. gratiosissimum* are nearly the same as those of *D. devonianum* (Table 6). Observed with excitation filter ex 530–590 nm and DM 595 nm emission filters, the fluorescence characteristics of *D. gratiosissimum* are similar to those of *D. officinale* (Table 6).

**Table 1:** Comparison of key microscopic characters of four species of *Dendrobium* (under normal light microscopy).

	40× *	100× *	200× *
*D. officinale*	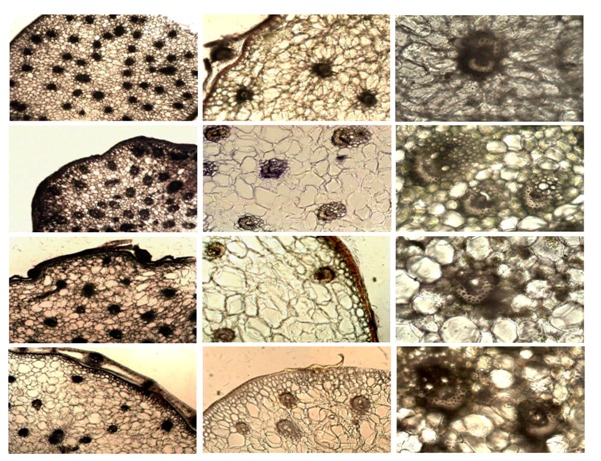		
*D. devonianu*			
*D* *.* * aphyllum*			
*D. gratiosissimum*			
	500 µm	250 µm	100 µm

* “×” stands for the amplification of the microscope.

**Table 2:** Comparison of microscopic features of *Dendrobium officinale*.

Sample No.	Cuticle	Epidermis	Fiber		Vascular bundles			Raphide
	Thickness	Diameter	Number	Diameter	Number	Diameter		Length
						Tangential	radial	
TP-1	9–12	14–34	17–29	8–19	78–90–102	73–125	85–229	65–68
TP-2	10–15	16–45	15–26	12–27	52–61–77	55–83	92–173	100–112
TP-3	8–13	12–29	16–37	10–28	84–92–108	61–118	80–187	75–79
TP-4	8–10	17–33	21–26	6–23	87–98–111	59–100	72–183	65–71
TP-5	8–10	13–26	20–58	8–24	84–88–108	67–104	90–236	100–103
TP-6	10–11	14–39	12–25	9–25	83–92–101	46–101	60–158	76–82
TP-7	9–11	12–37	14–22	11–29	54–69–86	42–140	51–147	59–96
TP-8	10–13	23–37	10–35	8–19	84–96–118	45–102	67–168	68–72
TP-9	9–10	17–27	20–30	5–18	85–92–103	45–89	50–135	53–92
TP-10	10–12	15–30	18–46	6–25	89–93–108	67–103	98–159	10–15
TP-11	10–18	13–38	18–33	6–20	79–93–99	50–102	63–169	146–194
TP-12	8–12	12–28	12–26	7–24	85–97–103	48–83	62–129	62–93
TP-13	10––12	12–36	11–46	7–31	61–65–97	56–119	73–196	62–156
TP-14	8–15	13–30	12–34	6–34	89–94–110	49–127	72–197	48–81
TP-15	9–21	11–39	9–31	8–37	93–109–134	54–145	71–237	52–190
Total	8–21	11–45	9–58	5–37	78(52) –134	42–145	50–237	10–194

**Table 3:** Comparison of microscopic features of *Dendrobium devonianum*.

Sample No.	Cuticle	Epidermis	Fiber		Vascular bundles			Raphide
	Thickness	Diameter	Number	Diameter	Number	Diameter		Length
						Tangential	Radial	
ZP-1	10–11	17–26	14–29	5–19	58–72–84	74–134	135–208	74–90
ZP-2	11–12	32–85	31–37	11–45	72–76–91	172–283	296–487	51–132
ZP-3	13–20	50–72	7–43	7–25	57–64–80	62–171	82–265	52–63
Total	11–20	17–85	7–43	7–45	57–91	62–283	82–487	51–132

**Table 4:** Comparison of microscopic features of *Dendrobium* *aphyllum*.

Sample No.	Cuticle	Epidermis	Fiber		Vascular bundles			Raphide
	Thickness	Diameter	Number	Diameter	Number	Diameter		Length
						Tangential	Radial	
SC-1	9–14	17–37	11–36	8–25	56–67–84	84–174	82–251	93–115
SC-2	14–16	13–30	20–47	5–33	64–72–85	88–127	99–281	65–182
SC-3	10–12	19–38	8–22	7–20	53–66–79	73–166	81–207	143–202
Total	9–16	13–38	8–47	5–33	53–85	73–174	81–281	65–202

**Table 5:** Comparison of microscopic features of *Dendrobium* *gratiosissimum*.

Sample No.	Cuticle	Epidermis	Fiber		Vascular bundles			Raphide
	Thickness	Diameter	Number	Diameter	Number	Diameter		Length
						Tangential	Radial	
GJ-1	8–10	11–32	20–66	6–31	57–65–124	104–346	89–218	53–124
GJ-2	17–18	15–33	23–52	6–29	59–72–81	108–293	92–173	39–73
GJ-3	16–17	17–35	19–31	7–24	68–71–84	127–278	102–225	51–71
Total	8–18	11–35	19–66	6–31	57–84(124)	104–346	89–225	39–124

**Table 6:** Comparison of key microscopic characters of four species of *Dendrobium* (under fluorescence microscopy).

	40× *		200× *	
	**B-1**	**G-1**	**B-1**	**G-1**
*D. officinale*	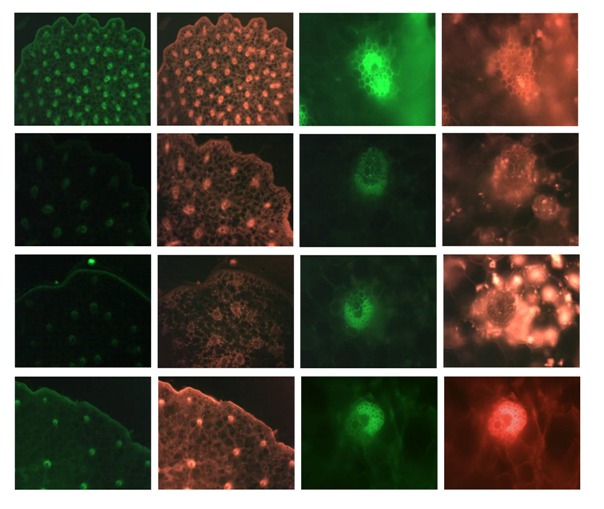			
*D.* *devonianum*				
*D. aphyllum*				
*D. gratiosissimum*				
	500 µm		100 µm	

B-1, emission filter of EF 450–500 nm; G-1, emission filter of EF 530–590 nm. * “×” stands for the amplification of the microscope.

### 2.3. Discussion

By combining normal light and fluorescence microscopy, *D. officinale* and the common adulterants can be easily differentiated from each other based on the characteristics of their transverse sections. Both types of microscopy have their own merits in the authentication study. Normal light microscopy can provide the basic morphological characteristics. As a supplemental tool, fluorescence microscopy can exhibit specific auto-fluorescence from different plant tissues by virtue of their varied chemical constituents, and it excels in manifestation of the shape (as shown in Table 6), which makes the authentication work more easy and convenient. The comparison of *D. officinale* and its adulterants can be summarized as follows:

(1) Cuticle: color and thickness of cuticle of four studied *Dendrobium* species are similar.
(2) Epidermis: size of epidermal cells of *D. officinale*, *D. aphyllum* and *D. gratiosissimum* are similar, epidermal cells of *D. devonianum* is bigger than the other three species, up to 85 μm in diameter.
(3) Vascular bundle: Vascular bundles of *D. officinale*, usually more than 90, are more abundant than in the three adulterants. The size of vascular bundles in *D. officinale* is similar apart from those near the epidermis which are slightly smaller. Vascular bundles of *D.* *devonianum* are about 70 in number, various in size, and much bigger in the centre than at the margins of the stem. Vascular bundles of *D. aphyllum* are about 65, with similar size, apart from those near to theepidermis that are slightly smaller. Vascular bundles of *D. gratiosissimum* are about 70, althoughmore than 100 can be observed occasionally. Various in size with no apparent distribution rules.
(4) Fiber groups: *D. officinale* with “two hat-shaped” fiber groups, while the other three species only have “one hat-shaped” fiber groups.
(5) Raphides: *D. officinale* scattered in parenchmatous cells near epidermis. *D. devonianum* scatteredin parenchmatous cells near epidermis and vascular bundles. *D. aphyllum* scattered in parenchmatous cells throughout. *D. gratiosissimum* scattered in parenchmatous cells near vascular bundles.

We can therefore put forth the following key to identifying *D. officinale* and its common adulterants:

(1). “Two hat-shaped” fiber groups emitted green fluorescence can be easily observed, Raphides distribute near the epidermi*s**D. officinale*(1). “One hat-shaped” fiber groups emitting green fluorescence can be easily observed
  (2). Vascular bundles similar in size, non-specific raphide distribution*D.*
*aphyllum*  (2). Vascular bundles various in size
    (3). Vascular bundles are much bigger in the centre than in the margin of the stem. Raphides distribute near the epidermis and vascular bundles*D.*
*devonianum*    (3). Vascular bundles of different size distribute with no obvious rule. Raphides distribute near the vascular bundle*D. gratiosissimum*

When fluorescence microscopy used, observed with excitation filter ex 450–500 nm and DM 505 nm emission filters, the cuticle, wall of epidermal cells and xylem vessels together with fiber groups outside vascular bundles emitted green fluorescence. Among them, the “two hat-shaped” fiber groups can be easily observed as the diagnostic feature of *D. officinale*. However, the other three *Dendrobium* species only possess “one hat-shaped” fiber groups. When observed with excitation filter ex 530–590 nm and DM 595 nm emission filters, though the red fluorescence can be observed in vascular bundles, it is not identical. Generally, xylem vessels in vascular bundles and fiber groups outside the vascular bundles emit red fluorescence, otherwise, the content of parenchymatous cells around the vascular bundles emit red fluorescence. Interestingly, the intensity and distribution of fluorescence are various in different samples, even of the same species, which may due to their different production areas, collection time and processing methods. Therefore, identification of the three adulterants only by fluorescence microscopy seems insufficient. The non-fluorescence microscopic features such as the distribution of rapheids, the size and amount of vascular bundles provided by the normal light microscopy were proposed to differentiate the three adulterants species.

## 3. Experimental Section

### 3.1. Materials

#### 3.1.1. Samples

Twenty-four samples comprising four species of *Dendrobium* stems were collected from different main production areas in China and Myanmar, and authenticated by Associate Prof. Hua-wei Zhang (Zhejiang University of Technology, Hangzhou, People’s Republic of China) and Prof. Zeng-xi Guo (Zhejiang Institute for Food and Drug Control, Hangzhou, People’s Republic of China). The details of each crude drug are given in Table 7. The voucher specimens were deposited in the Herbarium of Traditional Chinese Medicine, Zhejiang University of Technology.

**Table 7:** Crude drugs collected in markets and the main production areas.

Sample No.	Origin	Collection area/market	Collection date	Trade name
TP-1	*D. officinale*	GAP bases, Pu’er, Yunnan Province	September 2011	*Tie-pi-shi-hu*
TP-2	*D. officinale*	GAP bases, Tiantai, Zhejiang Province	October 2011	*Tie-pi-shi-hu*
TP-3	*D. officinale*	GAP bases, Tiantai, Zhejiang Province	September 2011	*Tie-pi-shi-hu*
TP-4	*D. officinale*	GAP bases, Tiantai, Zhejiang Province	August 2011	*Tie-pi-shi-hu*
TP-5	*D. officinale*	GAP bases, Tiantai, Zhejiang Province	April 2013	*Tie-pi-shi-hu*
TP-6	*D. officinale*	GAP bases, Tiantai, Zhejiang Province	May 2013	*Tie-pi-shi-hu*
TP-7	*D. officinale*	GAP bases, Tiantai, Zhejiang Province	Marrch 2013	*Tie-pi-shi-hu*
TP-8	*D. officinale*	Pan’an market, Zhejiang Province	September 2013	*Tie-pi-shi-hu*
TP-9	*D. officinale*	Pan’an market, Zhejiang Province (fresh)	September 2013	*Tie-pi-shi-hu*
TP-10	*D. officinale*	Pan’an market, Zhejiang Province (fresh)	September 2013	*Tie-pi-shi-hu*
TP-11	*D. officinale*	Wuyi, Zhejiang Province (fresh)	July 2013	*Tie-pi-shi-hu*
TP-12	*D. officinale*	Wuyi, Zhejiang Province (fresh)	July 2013	*Tie-pi-shi-hu*
TP-13	*D. officinale*	Pu’er, Yunnan Province (fresh)	July 2013	*Tie-pi-shi-hu*
TP-14	*D. officinale*	Pu’er, Yunnan Province (fresh)	July 2013	*Tie-pi-shi-hu*
TP-15	*D. officinale*	Pu’er, Yunnan Province (fresh)	July 2013	*Tie-pi-shi-hu*
ZP-1	*D. devonianum*	Myanmar (fresh)	January 2013	*Zi-pi-shi-hu*
ZP-2	*D. devonianum*	Pan’an market, Zhejiang Province	September 2013	*Zi-pi-shi-hu*
ZP-3	*D. devonianum*	Pan’an market, Zhejiang Province	September 2013	*Zi-pi-shi-hu*
SC-1	*D. aphyllum*	Myanmar (fresh)	January 2013	*Shui-cao-shi-hu*
SC-2	*D. aphyllum*	Pan’an market, Zhejiang Province	September 2013	*Shui-cao-shi-hu*
SC-3	*D. aphyllum*	Pan’an market, Zhejiang Province	September 2013	*Shui-cao-shi-hu*
GJ-1	*D. gratiosissimum*	Myanmar (fresh)	January 2013	*Guang-jie-shi-hu*
GJ-2	*D. gratiosissimum*	Pan’an market, Zhejiang Province	September 2013	*Guang-jie-shi-hu*
GJ-3	*D. gratiosissimum*	Pan’an market, Zhejiang Province	September 2013	*Guang-jie-shi-hu*

#### 3.1.2. Apparatus

(a) Optika digital camera DS-Fi1
(b) Optika Microscope equipped with CCD from Photometrics Coolsnap to capture photos.
(c) Optika Fluorescence Microscope B-600TiFL
(d) Canon digital camera 550D

#### 3.1.3. Software

Optika Vision Pro, TWAIN interface, SDK were used.

### 3.2. Method

#### 3.2.1. Morphological Characteristics of four *Dendrobium* Stems

The gross exterior characters of each sample were examined by observing, measuring, touching, smelling, and tasting. The color digital photographs were taken by Canon digital camera 550D.

#### 3.2.2. Transverse Section of Four *Dendrobium* Stems

The dried samples were moderately moistened firstly. Then, transverse section of samples (fresh sample or moistened dried sample) is made by cutting bare-handed. General procedures are as follows: three left fingers were used to fix the material at first. Then, put the blade which is held with the right hand against the material and slice smoothly from the left outward to the right inward. The tree-hand sections were investigated for possible auto fluorescence after sealing the mounted specimen along with purified water obtained from a Mili-Q water purification system (Millipore, Bedford, MA, USA).

**Figure 2:**
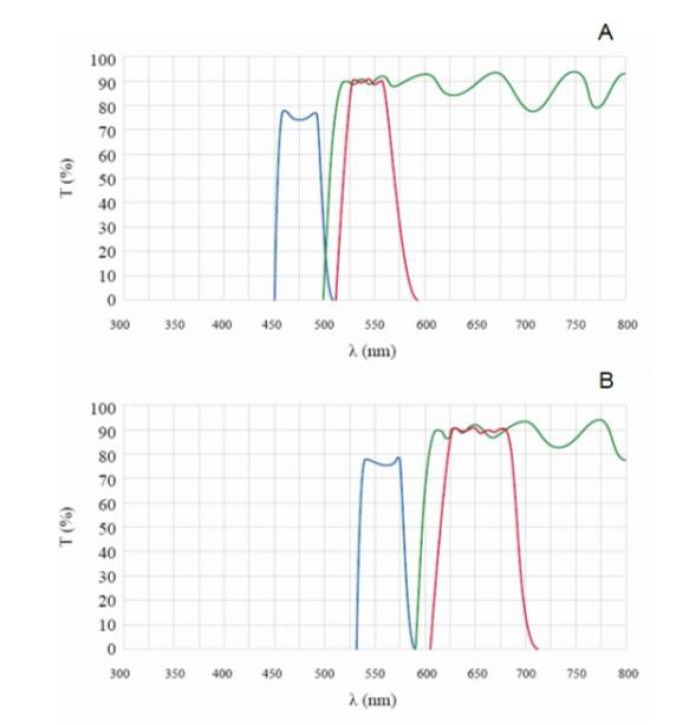
The transmittance spectra of fluorescence. (**A**) with EF 450–500 nm and DM 505 nm; (**B**) with EF 530–590 nm and DM 595 nm.

The images observed with normal light and fluorescence microscope were recorded digitally. The transmittance spectra of two emission filters are shown in Figure 2. Excitation from HBO 100 light source through the excitation filter (EF) 450–500 nm and emission through dichroic mirror (DM) 505 nm (blue light), as well as excitation through filter BP 530–590 nm and emission through dichroic mirror DM 595 nm (green light) were used for observing fluorescence.

The blue line is the excitation wavelength; the red line stands for the barrier filter wavelength; the green line stands for the dichroic mirror cut-off wavelength.

## 4. Conclusions

This is the first research combining normal light and fluorescence microscopy to thoroughly identify *Dendrobium officinale* and its counterfeits sold in the market through an investigation of the transverse sections of crude drug stems. Fluorescence microscopy, as a supplementary tool for routine microscopic identification, provides *in vivo* pictures and needs no professional researchers. According to the fluorescence characteristics, a further histochemical investigation on the chemical distribution in different tissues of *D.* *officinale* will be carried out. It is expected to be of great use in the identification and quality evaluation of *Dendrobium* species.
